# Editorial: Refugee Mental Health

**DOI:** 10.3389/fpsyt.2019.00072

**Published:** 2019-02-26

**Authors:** Stephan Zipfel, Monique C. Pfaltz, Ulrich Schnyder

**Affiliations:** ^1^Department of Psychosomatic Medicine and Psychotherapy, University of Tübingen, Tübingen, Germany; ^2^Department of Consultation-Liaison Psychiatry and Psychosomatic Medicine, University Hospital Zurich, University of Zurich, Zurich, Switzerland; ^3^University Hospital Zurich, University of Zurich, Zurich, Switzerland

**Keywords:** refugees, asylum seekers, migration, trauma, PTSD, refugee mental health

Humans have been migrating across the globe since they first occurred in evolution millions of years ago (1). The reasons for human migration were, and still are, manifold: draughts and other ecological changes; curiosity and the drive to explore the world; plagues; population growth; economic hardship and hope for a better life; struggle for power and dominance; etc. However, fleeing from warfare, persecution and suppression has kept constituting an important, and particularly unpleasant driving force over the centuries: many people leave their habitat and migrate within their country or across the border to escape life-threatening, humiliating, or otherwise unbearable situations.

The ever growing numbers of migrants globally are a challenge to humankind. An estimated one billion people are on the move currently, of which about 65–70 million have been forcibly displaced (2, 3). This is a phenomenon of fundamental relevance to our society. It challenges our identities and our value systems, and we are more and more urgently confronted with questions that will not be easy to respond to: What is it that constitutes my identity? Since in many cases, it is no longer the place where I was born and raised, and the language my parents spoke, what is it? And given that an increasing number of people who live in my neighborhood do not share my cultural values, what is actually at the core of my value system?

According to the 1951 Refugee Convention of the United Nations High Commissioner for Refugees (UNHCR), a refugee is “a person who, owing to well-founded fear of being persecuted for reasons of race, religion, nationality, membership of a particular social group or political opinion, is outside the country of his nationality and is unable or, owing to such fear, is unwilling to avail himself of the protection of that country” (4).

The term “refugee mental health” has been established to describe mental health issues related to various aspects of becoming, being, or having been a refugee, such as traumatic exposure in one’s home country that led to the person’s flight, adverse experiences during the flight, as well as the various challenges refugees are frequently exposed to post-flight, when trying to integrate in their host country’s society. Of note, from a mental health perspective, refugee mental health does not apply to refugees in a legal sense exclusively but equally to asylum seekers and internally displaced persons, i.e., persons who have fled their homes but never crossed an international border.

*Frontiers in Psychiatry* is a journal for mental health professionals, and we as guest editors are in no position to find answers to fundamental societal questions that are beyond the range of our professional expertise and experience. We therefore focused on mental health issues in various refugee populations, hoping to make a small contribution to humankind’s attempt at dealing with an extremely complex phenomenon of which mental health is but one of many ramifications. Refugee mental health constitutes a major public mental health issue (5). Additionally, given the strong associations between trauma exposure, trauma-related psychopathology, and physical health (6), we also need to view refugee mental health as a public health issue in a much broader sense.

Refugee mental health is understudied for various reasons. First and foremost, there is the language barrier. The fact that the costs for professional translators to facilitate mental health service provision are not covered in virtually all, and even the most highly developed healthcare systems worldwide, is no less than a scandal. Many asylum seekers and refugees don’t speak the locally spoken language of their host country. Using professionally trained translators is not only cost-intensive and time-consuming, and thus burdens research budgets. It also raises questions about the reliability of psychometric data (7, 8). This being said, without being able to communicate adequately, we will never be able to conduct meaningful research, nor will it be possible to provide effective treatment. Secondly, studying traumatized refugees’ mental health means to take into account cross-cultural aspects which adds to the complexity of this field of research but also makes it particularly interesting (9). When collecting a heterogeneous clinical sample of people from various cultural backgrounds, issues of representativity need to be dealt with. Conversely, when studying a group of people with the same ethnicity, race, or religious beliefs, generalizability will be very limited as well.

We still know very little about how evidence-based concepts of assessing and treating mental health conditions actually work when applied to traumatized refugee populations. This is particularly true with regard to children and adolescents, especially when they are unaccompanied by their caregivers, and maybe even more so with regard to elderly refugees. Also, the interplay between pre-migration adverse or potentially traumatic experiences, various stressors during the flight, post-migration living difficulties, and mental health is far from being understood. Assessment and treatment of mental health conditions in traumatized refugees may require specific cultural adaptations. There is also a great need to better understand the link between mental health and refugees’ integration in their host countries’ societies.

This is why we launched the research topic (RT) on refugee mental health. In a total of 14 contributions to our RT, we deal with different target groups. The spectrum ranges from asylum seekers (Womersley and Kloetzer) to the largest group of refugees and post-conflict survivors (e.g., Killikelly et al.) to the group of immigrants (Lanzara et al.) There are papers with a special focus on the group of children and the question of their mental health status (Buchmüller et al.) as well as research results on the group of extremely traumatized children and adolescents and their mothers from the group of Yazidi people who have become victims of extreme violence by the so-called Islamic State (Rometsch-Ogioun El Sount et al.). In addition to the findings in the Yazidis, from which the Nobel Peace Prize winner of 2018 Nadia Murat emerged, there are also contributions on possible secondary traumatization in caregivers (Denkinger et al.; Rometsch-Ogioun El Sount et al.). With regard to the geographical fields of conflict, there are not only contributions from northern Iraq but also from the “hot spots” Syria (Georgiadou et al.) and East Africa (Schneider et al.). The spectrum of contributions also includes systematic reviews (Killikelly et al.; Lanzara et al.; Morina et al.), assessments of mental health and distress (e.g., Nickerson et al.; Kaltenbach et al.) as well as initial intervention studies in group format for traumatized male refugees (Zehetmair et al.) or app-based e-mental health interventions for Syrian refugees (Burchert et al.).

We would like to thank all colleagues who, by submitting their manuscripts and sharing their expertise and experiences in this highly relevant but also difficult field, make it possible for us as both psychotherapists and researchers to keep up with the dramatic developments of recent years and to develop evidence-based, effective strategies and interventions for the highly stressed group of forcibly displaced persons as an important step toward faster and better integration into their new home countries (Schick et al.).

## Author Contributions

All authors listed have made a substantial, direct and intellectual contribution to the work, and approved it for publication.

### Conflict of Interest Statement

The authors declare that the research was conducted in the absence of any commercial or financial relationships that could be construed as a potential conflict of interest.
